# Nitric Oxide Synthase Inhibition Enhances the Antitumor Effect of Radiation in the Treatment of Squamous Carcinoma Xenografts

**DOI:** 10.1371/journal.pone.0020147

**Published:** 2011-05-25

**Authors:** Robert J. G. Cardnell, Ross B. Mikkelsen

**Affiliations:** Department of Radiation Oncology, Virginia Commonwealth University, Richmond, Virginia, United States of America; National Cancer Institute, United States of America

## Abstract

This study tests whether the nitric oxide synthase (NOS) inhibitor, N_G_-nitro-L-arginine (L-NNA), combines favorably with ionizing radiation (IR) in controlling squamous carcinoma tumor growth. Animals bearing FaDu and A431 xenografts were treated with L-NNA in the drinking water. IR exposure was 10 Gy for tumor growth and survival studies and 4 Gy for *ex vivo* clonogenic assays. Cryosections were examined immunohistochemically for markers of apoptosis and hypoxia. Blood flow was assayed by fluorescent microscopy of tissue cryosections after i.v. injection of fluorospheres. Orally administered L-NNA for 24 hrs reduces tumor blood flow by 80% (p<0.01). Within 24 hrs L-NNA treatment stopped tumor growth for at least 10 days before tumor growth again ensued. The growth arrest was in part due to increased cell killing since a combination of L-NNA and a single 4 Gy IR caused 82% tumor cell killing measured by an *ex vivo* clonogenic assay compared to 49% by L-NNA or 29% by IR alone. A Kaplan-Meyer analysis of animal survival revealed a distinct survival advantage for the combined treatment. Combining L-NNA and IR was also found to be at least as effective as a single i.p. dose of cisplatin plus IR. In contrast to the *in vivo* studies, exposure of cells to L-NNA *in vitro* was without effect on clonogenicity with or without IR. Western and immunochemical analysis of expression of a number of proteins involved in NO signaling indicated that L-NNA treatment enhanced arginase-2 expression and that this may represent vasculature remodeling and escape from NOS inhibition. For tumors such as head and neck squamous carcinomas that show only modest responses to inhibitors of specific angiogenic pathways, targeting NO-dependent pro-survival and angiogenic mechanisms in both tumor and supporting stromal cells may present a potential new strategy for tumor control.

## Introduction

Nitric oxide synthase (NOS) activity is a key component in a number of survival mechanisms integrated into the autocrine and paracrine nature of tumor cells and supporting stromal cells. For example, the catalytic activities of protein tyrosine phosphatases such as SHP-2 that modulate the receptor tyrosine kinases (RTK) are modulated by S-nitrosylation and oxidation of their active site cysteine [1], [2]. The basal activities of other key regulatory proteins such as the transcription factor, NF-κB, are also sensitive to nitro-oxidative stress. NF-κB and as a consequence a number of its target cytoprotective genes are constitutively activated in numerous cancers including head and neck squamous cell carcinomas (HNSCC) cell lines and tissues [3], [4], [5]. *In vitro* experiments with different cell types have demonstrated that 30–50% of the basal NF-κB activity is sensitive to either NOS inhibitors or dominant negative NOS mutants [6].

These NOS-dependent survival mechanisms are also activated by ionizing radiation (IR). For example, (IR) stimulates the activity of eNOS (NOS-3) in tumor endothelial cells resulting in enhanced tumor angiogenesis through RTK-dependent and –independent mechanisms [7], [8], [9]. IR also stimulates NOS activity in tumor cells activating diverse anti-apoptotic mechanisms involving RTK and NF-κB signaling pathways. In mutant Ras transformed cells, Akt phosphorylation and activation of eNOS results in the S-nitrosylation (or oxidation to sulfenic acid) of Ras Cys118, enhancing GTP binding, and thereby stimulating cytoprotective signaling pathways [10]. *In vitro* experiments have shown that activation of these NF-κB and RTK “pro-survival” mechanisms by IR can be inhibited by the NOS inhibitor N^G^-nitro-L-arginine (L-NNA) [6], [7].

Previous studies of fibrosarcoma type II and hepatocarcinoma transplantable liver tumors have shown that the L-NAME (the bio-inactive pro-drug of L-NNA) has no effect upon short term tumor oxygenation following 4 Gy IR but inhibits an increase in tumor pO_2_ observed 24 hours post irradiation [11], [12]. Short-term administration of L-NAME also does not add to the delay of tumor growth seen with a single dose of IR [11], [13]. These and similar studies have not, however, studied the effects of long term NOS inhibition upon tumor growth or cell killing, nor have they utilized the active drug, L-NNA. This fully active NOS inhibitor, L-NNA, selectively reduces the blood flow to P22 carcinosarcomas in BD9 rats [7], [14]. Furthermore, a clinical phase I dose escalation study demonstrated that a single i.v. dose of L-NNA decreases tumor vascular blood volume by 40%, an effect that is sustained 24 hours post-treatment with minimal side effects (toxicity level 1) [15].

Recent studies have also examined whether the anti-tumor activity of the vascular disrupting agent, combrestatin A-4 3-O-phosphate is enhanced by the co-administration of L-NNA [16], [17]. The combination of the two vascular targeting agents achieved therapeutic enhancement over either agent alone as measured by tumor growth delay. The combination of two systemic anti-vascular agents is potentially very toxic to normal tissues. For this reason we investigated whether L-NNA treatment interacts favorably with a second targeted anti-tumor therapy, radiation. From a radiobiological perspective, radioresistance is associated with acute hypoxia. However, prolonged or chronic hypoxia (>72 hr) has been shown in some *in vitro* studies to enhance cellular radiosensitivity by a mechanism involving inhibition of cell energy metabolism and as a consequence inhibition of DNA repair mechanisms [18], [19]. Here we report that the addition of L-NNA to drinking water of tumor-bearing animals delays tumor growth, elicits tumor cell killing through apoptosis, preferentially reduces tumor blood volume and, when combined with a single 10 Gy IR dose, enhances animal survival. These observations suggest that anti-NOS agents in combination with IR can be therapeutically beneficial in the treatment of squamous carcinomas.

## Methods

### Cell culture

A431 and FaDu cells from the American Type Culture Collection (Manassas, VA) were maintained in RPMI 1640 medium supplemented with 10% fetal bovine serum at 5% carbon dioxide in a humidified 37°C incubator. For hypoxia studies cells were plated under normoxic conditions and subsequently moved to a hypoxic glove box (Coy Laboratory Products, Inc) where they were equilibrated at low oxygen levels for 1 hour before being placed in billups-rothenburg chambers incubated at 37°C. *In vitro* clonogenic assays followed protocols established elsewhere [19].

### Mouse xenograft model

Tumor xenografts were created by the subcutaneous injection of 10^6^ A431 or 5×10^5^ FaDu cells suspended in sterile saline into the hind flanks of 5–6-week-old athymic NCr-nu/nu male mice. Tumor dimensions were calculated three times weekly from caliper measurements of the tumor made using the formula width^2^×length×0.4 (where the width is the smaller dimension). The mice designated for treatment with the NOS inhibitor were provided with drinking water supplemented with 0.5 g/L L-NNA (Sigma-Aldrich, St. Louis, MO). Fresh L-NNA-containing water was provided three times weekly. Mice were obtained from the National Cancer Institute (Frederick, MD). Animals designated for cisplatin treatment received a single 8 mg/kg in sterile saline bolus i.p. on day 0 prior to initiation of L-NNA treatment, but no more than 24 hours prior to irradiation [20]. All procedures were approved by the Institutional Animal Care and Use Committee of Virginia Commonwealth University and conformed to the guidelines established by the National Institutes of Health, protocol numbers AM10080 and AM10185.

### *In vivo* radiation treatment

Mice were anaesthetized with ketamine/xylazine. The irradiation beam from a clinically retired Picker ^60^Co source was focused on the posterior legs to minimize irradiation of the abdomen. Radiation exposure was 10 Gy for tumor growth and animal survival assays. For the *ex vivo* clonogenic assay the single radiation dose was 4 Gy.

### *Ex vivo* clonogenic cell survival assay

Twenty-four hours post-irradiation or post L-NNA-treatment, tumors were harvested and cells derived therefrom were plated for colony formation assays as previously described [21].

### Blood flow analysis

When A431 xenografts reached the size of approximately 150 mm^3^, half the mice received drinking water supplemented with L-NNA. Twenty-four hours later 0.25 ml of 0.1 µm diameter blue FluoSphere beads (Invitrogen, Carlsbad, CA) diluted 1∶6 in physiological saline was delivered by tail vein injection [22]. Three minutes post-injection the animals were sacrificed and the tumor, heart, liver, spleen and kidney excised, flash frozen and embedded in OCT (Sakaru, Torrance, CA) for cryosectioning. Cryosections of 8 µm thickness were examined by direct fluorescent microscopy at 10× magnification. Five randomly selected frames from each cryosection were quantified using ImageJ (NIH).

### Immunohistochemistry

Tumors were excised from animals and immediately flash frozen in liquid N_2_, embedded in OCT and 6 µm cryosections prepared. Apoptosis was assayed using the Apoptag™ (Millipore, Billerica, MA) kit as per the manufacturer's instructions.

Areas of hypoxia were identified and quantified by three staining procedures (exogenous pimonidazole and endogenous HIF1α and CA-9 expression). Pimonidazole HCl (HPI Inc., Burlington, MA) was injected i.p. (200 mg/kg in sterile saline). At 3 hours post injection tumors were excised, frozen, and prepared for immunohistochemical analysis. Cryosections were fixed in 4% paraformaldehyde, permeabilized in 0.25% Triton, endogenous peroxidase quenched with 3% H_2_O_2_ in 10% methanol, and blocked with 10% normal donkey serum. Immunostaining was performed by overnight incubation with rabbit anti-pimonidazole antisera (PAb2627, Hydroxyprobe, Inc.), rabbit anti-carbonic anhydrase IX (1∶100, ab15068, AbCam, Cambridge, MA) and rabbit anti-HIF1α (Pre-diluted, ab76471, AbCam) primary antibodies. An HRP conjugated anti-rabbit secondary antibody (2 µg/ml, Rockland Immunochemicals, Gilbertsville, PA) followed by 5 minute 3,3′-Diaminobenzidine (DAB) was used to indicate hypoxic cells; hemotoxylin was used as a counterstain. Images of stained sections were captured and analyzed using the Ariol high throughput histopathology system (Genetix USA, Inc., Boston, MA). The Ariol system quantifies positive staining using color and shape classifiers to analyze the entirety of each section imaged. Analysis with the classifiers can be used to count shapes (e.g. nuclei) of predetermined sizes and colors or areas of particular colors allowing for calculations of percentage of nuclei that stain positive or area that stains positive. The threshold for positive staining was established using sections stained without the primary antibody.

For Hematoxylin and Eosin staining, cryosections (10 µm) were fixed in 4% paraformaldehyde, submerged in Modified Mayer's Hematoxylin (Richard Allan Scientific), destained in acid ethanol, stained with eosin, dehydrated and mounted with permount. Images were captured and analyzed using the Ariol system.

### Western blot

Protein was extracted from flash frozen tumors using a pestle and mortar and 20 ml/g tumor RIPA lysis buffer (50 mM Tris-HCL pH 7.5, 10 mM NaCl, 1% Triton X-100, 1% sodium deoxycholate, 0.1% SDS, 5 mM EDTA) and processed under reducing conditions. Proteins were resolved with 8% resolving SDS-polyacrylamide gel electrophoresis and transferred to nitrocellulose membranes. Membranes were incubated with dilutions of 1∶1000 of arginase II, iNOS or actin antibodies (Santa Cruz Biotechnology, Santa Cruz, CA) overnight at 4°C. Blots were developed using IR 700 or IR 800 conjugated secondary antibodies (Rockland Immunochemicals, Gilbertsville, PA) diluted 1∶10,000 and imaged/analyzed using the Odyssey Licor system.

### Statistics

Statistical significance calculated using un-paired student t-tests for all assays other than animal survival where log-rank analysis was performed. Thresholds for statistical significance are indicated in the text. Variances are presented as the standard error of the mean. “N” values indicate the number of animals used; “n” values the number of tumors.

## Results

### L-NNA delays A431 and FADU xenograft growth

**Figure 1:**
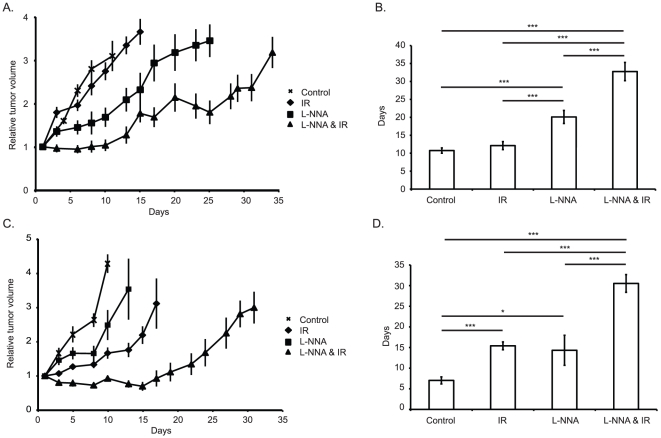
L-NNA and IR reduce the rate of xenograft growth. Relative volumes of differently treated A431 (A) and FaDu (C) xenografts. Mean tripling time of A431 (B) and FaDu (D) xenografts. Xenografts were created in athymic nu/nu mice by subcutaneous injection of 10^6^ A431 or 5×10^5^ FaDu cells into the hind flank, once tumors were established animals were divided into 4 groups as indicated. L-NNA was provided from day 0 at 0.5 g/L in drinking water, irradiated animals received a single 10 Gy dose targeted at the flank using a ^60^Co source on day 1. Data presented as mean relative volume ± SEM or mean days to triple volume ± SEM; N = 5, n = 10. * = p<0.05, *** = p<0.01. Horizontal lines and accompanying asterisks indicate statistically significant differences.

To determine the effect of L-NNA on the growth of tumors, xenografts were grown unhindered until approximately 150 mm^3^ in volume, at which point they were divided into four treatment groups: control; 0.5 g/L L-NNA in drinking water from day 0; 10 Gy IR on day 1; or L-NNA from day 0 and 10 Gy IR on day 1. Tumor growth delay was determined by the average tumor volume relative to treatment day 0 for each group (Fig. 1A) and by the time for the tumor volume to triple relative to treatment day 1 (Fig. 1B). Both the L-NNA and L-NNA plus IR treatments were effective in delaying A431 tumor growth compared to control non-treated and irradiated alone tumors. The combined treatment caused a growth delay significantly longer than either treatment alone.

A similar set of experiments was performed with FaDu xenografts to evaluate if the inhibitory effect of L-NNA on tumor growth could be generalized to another tumor. Tumor growth was assayed by average tumor volume (Fig. 1C) and tripling time (Fig. 1D). All three treatments delayed tumor growth compared to control with the combined treatment being the most effective. The delay observed in FaDu xenografts was greater than that observed in A431 xenograft-bearing animals.

We also tested the methyl ester pro-drug of L-NNA, *N*ω-nitro-L-arginine-methyl ester (L-NAME). L-NAME (0.5 g/L in drinking water) elicits a much shorter delay in the growth of both A431 and FaDu xenografts (49% and 27% increase in tripling time over control respectively). L-NAME must be hydrolyzed to L-NNA in order to become bioactive and this requirement for processing may limit bioavailability of L-NNA [23].

### L-NNA plus IR increases survival

**Figure 2:**
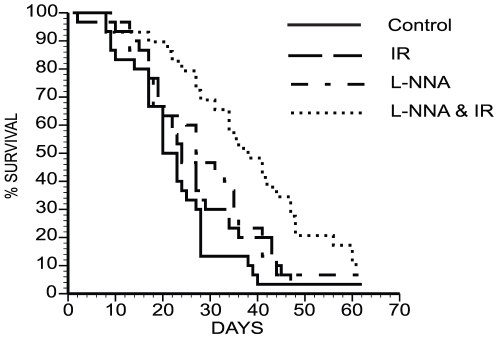
L-NNA with IR enhances animal survival. Survival of differently treated FaDu xenograft bearing mice. Kaplan-Meyer survival plot of animals receiving either no treatment; 10 Gy IR; L-NNA; or L-NNA and 10 Gy IR. Animals receiving L-NNA and IR survived significantly longer than all other groups as determined by log-rank analysis (p<.05, N≥29).

During the analysis of the growth of FaDu xenografts, it became apparent that animals receiving both L-NNA and IR outlived their counterparts receiving either no or mono-therapies. As shown in Figure 2 the combined L-NNA plus IR treated animals survived significantly longer than the control, IR only or L-NNA only groups. Median survival was increased to 37.5 days in animals receiving both L-NNA and IR as compared to 20 days (p = 0.00007) for control animals, 23.5 days (p = 0.003) for animals receiving only IR and 27 days (p = 0.26) for the L-NNA alone group. To further quantify the significance of the combined treatment we calculated the enhancement factor [24]. Enhancement factors of greater than 2.5 were determined for both FaDu xenograft tripling time and animal survival indicating the combined L-NNA and IR treatment to be more than the additive effect of the individual treatments. While a reduced tumor burden undoubtedly plays a role in enhanced survival, that either IR or L-NNA alone is sufficient to reduce the rate of FaDu xenograft growth but not to significantly enhance survival suggests other mechanisms might be involved. Possible mechanisms are discussed more fully below.

### L-NNA induces tumor cell killing

**Figure 3:**
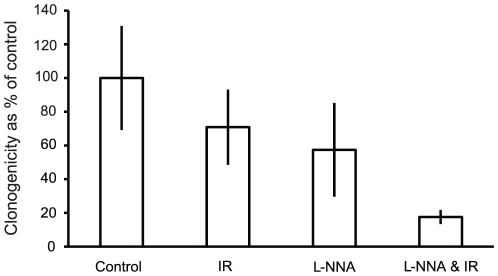
L-NNA reduces cell survival. Clonogenic survival of A431 cells isolated from flank xenografts following the treatments indicated. Data presented as mean colonies formed as a percentage of control ± SEM; N = 3, n = 6.

Having shown L-NNA to slow xenograft growth and L-NNA plus IR to enhance survival, *ex vivo* clonogenic assays with A431 xenografts were performed to determine what proportion of tumor cells are killed within the first 24 hrs of each treatment. As shown in Figure 3, 4 Gy IR or overnight L-NNA alone result in 29% and 49% respectively fewer colonies formed as compared to control. The combination of overnight L-NNA consumption followed by IR, however, elicited an 82% reduction in the number of colonies formed compared to untreated controls. These results show L-NNA by itself caused significant tumor cell killing compared to untreated controls *in vivo*. However, *in vitro* clonogenic assays using A431 monolayers exposed continuously to L-NNA concentrations up to 100 µM failed to show any decrease in clonogenicity. This *in vitro* finding suggests that other mechanisms possibly involving the tumor cell microenvironment come into play for L-NNA to have it cytotoxic effects on the tumor cells *in vivo*.

### L-NNA treatment induces apoptosis

Since it has been shown that L-NNA inhibits basal and IR-induced anti-apoptotic signaling pathways such as NF-κB [6], we tested whether L-NNA treatment enhanced apoptosis *in vivo*. Cryosections from tumors following 0 and 1 day of L-NNA treatment were stained by immunohistochemistry for 3′OH DNA terminus as a measure of apoptosis. Analysis of sections from multiple experiments shows a significant increase in the number of cells staining positive for apoptosis following 1 day of L-NNA treatment. At Day 1 a 4.4 fold (±1.7, p = 0.04) increase over day 0 in the proportion of apoptotic cells is observed. The increased apoptosis provides one explanation for the rapid induction of growth delay seen with L-NNA treatment (Figure 1A).

### L-NNA preferentially reduces tumor blood flow

**Figure 4:**
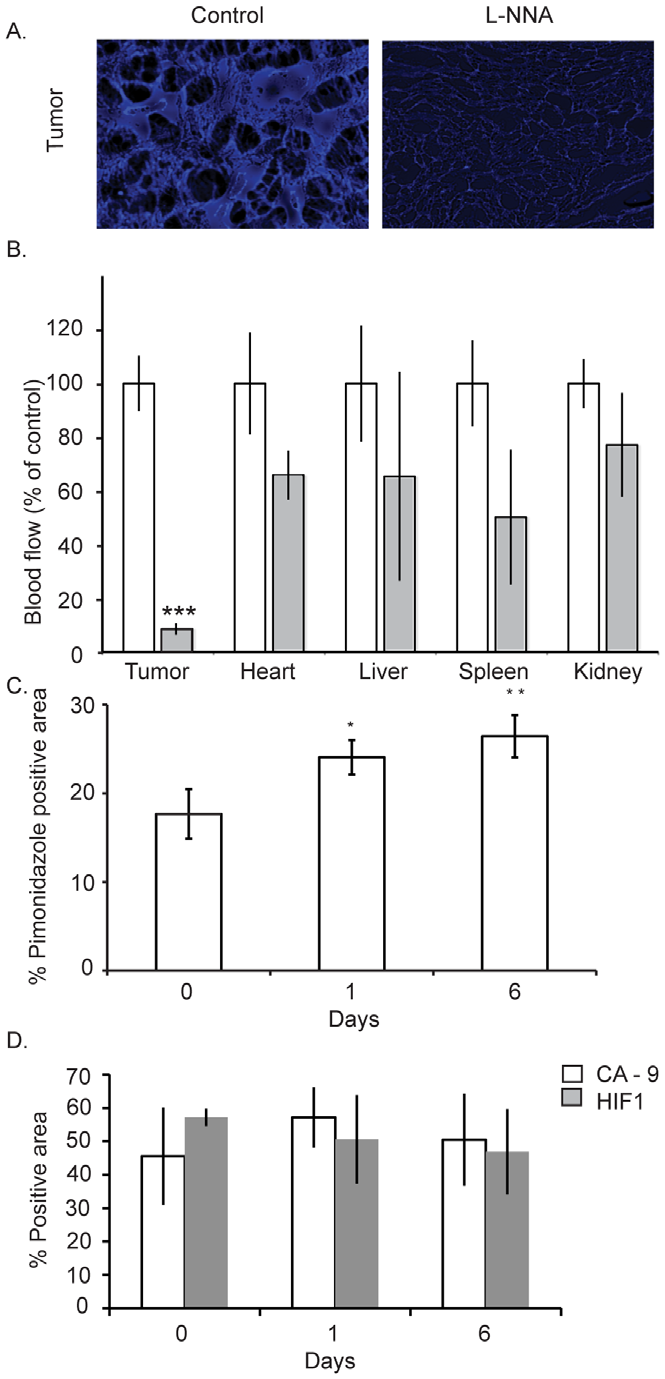
L-NNA treatment decreases tumor blood flow and increases hypoxia. Comparison of tissue vasculature from L-NNA treated and control mice. Example direct fluorescent images from control and L-NNA treated A431 tumors (A). Quantification of decreased blood flow to tumors and other tissues following L-NNA treatment (B). Blood flow was determined by tissue penetration of i.v. injected fluorescent beads within 3 minutes of injection. Data presented as mean intensity as a percentage of control ± SEM; n = 5; *** = p<0.01. A431 tumor hypoxia following L-NNA treatment measured immunohistochemically by pimonidazole (C), carbonic anhydrase IX and HIF-1α expression (D).

In both humans and rats intravenous introduction of L-NNA induces a reduction in tumor blood flow [14], [15]. We tested whether this also occurs in mice with L-NNA delivered in the drinking water for 24 hours. Tissues from animals bearing a single A431 flank xenograft were excised 3 minutes after tail vein injection of fluorescent beads and subsequently analyzed by direct fluorescent microscopy to determine the degree to which the beads had penetrated each tissue (Figure 4). Whereas L-NNA causes some reduction in blood flow in all tissues analyzed, the only statistically significant decrease is in the tumor (>80%, p<0.01).

We tested the possibility of increased tumor hypoxia with immunohistochemical analysis for markers of hypoxia in tumors following 0, 1 and 6 days of L-NNA treatment. Pimonidazole, carbonic anhydrase IX (CA-9) and HIF1α are established markers of hypoxia. Figure 4 shows the proportion of cells staining positive for pimonidazole to increase significantly following L-NNA treatment (17.7% to 24.1%, p<0.05, Figure 4C) suggesting that the hypoxic volume of the tumors is increased. The CA-9 and HIF1α markers (Figure 4D) do not show a significant increase following L-NNA treatment. As discussed below the insensitivity of the endogenous hypoxia markers may reflect their low dynamic response range to oxygen concentration compared to pimonidazole and their complex responses to tumor microenvironment including NO.

**Figure 5:**
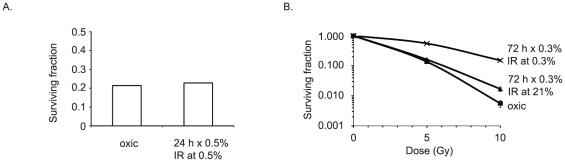
Chronic hypoxia and radiosensitivity *in vitro*. A431 cells cultured under normoxic and chronic hypoxic condition show decreased sensitivity to irradiation following long-term hypoxia. 24 hour hypoxic incubation (0.5% O_2_) prior to 4 Gy irradiation has no effect upon cell survival (A). Chronic (72 hour) hypoxia decreases radiosensitivity at 5 and 10 Gy, but reoxygenation for 6 hours prior to IR restores radiosensitivity (B). Data presented as mean surviving fraction ± SEM. In all cases cells were plated in triplicate at two cell densities.

Based on the results with pimonidazole we tested the effect of chronic hypoxia (>72 hrs) on the radio-sensitivity of A431 cells *in vitro*. We expected, based on previous reports, that chronic hypoxia, as opposed to acute hypoxia, might actually radiosensitize [18], [19]. However, as shown in Figure 5B, similar long-term hypoxic conditions with A431 cells actually induced radio-resistance *in vitro*, whereas acute hypoxia had no effect on radiosensitivity (Figure 5A). These results suggest the effects of L-NNA *in vivo* are not simply the result of hypoxia.

### Arginase II but not iNOS expression increases following L-NNA treatment

**Figure 6:**
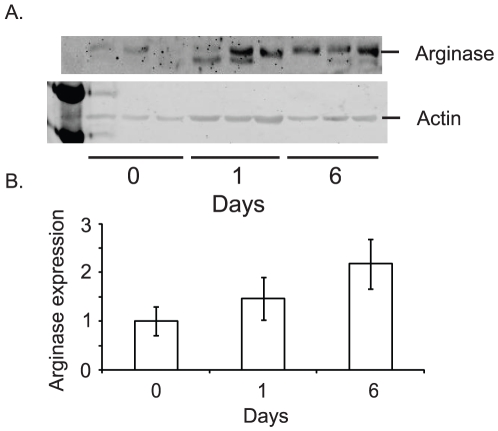
Arginase II expression increases following L-NNA exposure. Western blots of arginase II and actin expression in A431 xenografts prior to and following 1 and 6 days of L-NNA treatment (A). Quantification of relative arginase II expression standardized to actin (B). Data presented as mean ± SEM; n = 3, * = p<0.05.

L-arginine, the substrate for NOS, is also metabolized by arginase to produce urea and L-ornithine. It has been proposed that a switch from the metabolism of arginine by NOS to arginase is part of the transition of the inflammatory response from the early NO-driven “anti-bacterial” phase to the later polyamine dependent repair phase [25], [26]. Arginase II expression increases in pulmonary arterial smooth muscle cells under hypoxic conditions [27]. The upregulation of arginase II expression has also been implicated in smooth muscle cell growth and collagen synthesis, suggesting a role in aberrant vessel wall remodeling [27]. Given these findings and that the inhibition of NOS is likely to result in an accumulation of L-arginine, we determined the expression of arginase II in A431 xenografts prior to and following L-NNA treatment. As shown in figure 6, there is little arginase II expressed prior to L-NNA treatment. Expression increased starting 1 day post treatment with L-NNA and continuing with a statistically significant increase observed at day 6. Immunohistochemical analysis of tumor sections for arginase II expression showed expression in a majority of cells at all three time points (data not shown). Since most cells express arginase II, the observed increase in arginase II expression is due to either increased expression or decreased degredation in individual cells rather than an increase in the number of cells that express arginase II. The timing of the increased arginase II expression may be particularly pertinent. Arginase catalyzes arginine to ornithine conversion, an important step in polyamine synthesis and which has been shown to promote tumor growth and survival [28].

A previous *in vitro* study with EMT-6 tumor cells demonstrated increased expression of iNOS following 24 hours of 1% O_2_ [29]. Western blot analysis of A431 tumor lysates for iNOS showed minimal basal expression in the xenografts and no meaningful change in iNOS expression with L-NNA treatment.

### L-NNA treatment is comparable to single bolus cisplatin treatment

**Figure 7:**
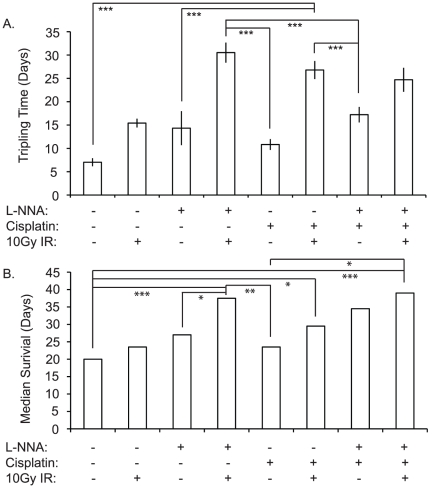
L-NNA treatment is comparable to cisplatin. L-NNA with IR is seen to be as, or more, effective than any other treatment in A431 xenografts. Flank xenograft tripling time following the treatments indicated (A). Data presented as mean ± SEM. Median survival times of animals bearing flank xenografts following the treatments indicated (B). Significance calculated by log-rank analysis. Cisplatin delivered as a single i.p. bolus at 8 mg/kg on day 0; L-NNA provided from day 0 at 0.5 g/L in drinking water; irradiated animals received a single 10 Gy dose targeted at the flank using a ^60^Co source on day 1. (* = p<0.05, ** = p<0.02, *** = p<0.01).

We compared the effectiveness of L-NNA treatment relative to, and in combination with, cisplatin. Flank FaDu xenograft-bearing animals were treated with either: cisplatin; cisplatin and IR; cisplatin and L-NNA; or cisplatin, L-NNA and IR. A treatment modality whereby animals received a single large bolus i.p. of cisplatin was used as this modality has been shown to effective *in vivo* and was most compatible with our existing L-NNA and IR treatment plan [20]. The animals were monitored for tumor volume and survival. As seen in Figure 7, the delay in tripling of the tumor volume and the enhancement of survival observed following L-NNA with IR is equal to the effect of single bolus cisplatin with IR. Interestingly, the triple treatment provided no significant advantage over either L-NNA and IR or single bolus cisplatin and IR; indeed the triple treatment was highly toxic with 30% mortality by day 10 post-treatment.

## Discussion

Analyses of tumor growth delay, animal survival and *ex vivo* clonogenicity show that L-NNA when combined with IR to be highly effective in controlling squamous carcinoma xenografts. L-NNA alone is not without benefit and is as effective as a single 4 Gy IR dose when evaluated by *ex vivo* clonogenic assay 24 hrs post treatment. L-NNA by itself kills tumor cells *in vivo*, in part through promoting apoptosis. It is, however, not entirely clear if increased tumor cell apoptosis accounts for the total effect of L-NNA. Differentiation of some tumor cells can also explain the results observed and A431 and FaDu cell types are prone to differentiation through cornification, particularly following IR [24], [30]. Histopathological analysis of L-NNA treated tumors, however, shows no increase in differentiation. Similarly, no significant increase in areas of necrosis is observed following L-NNA treatment. A previous study also did not observe increased necrosis with L-NNA treatment [16]. The decreased blood flow to the tumor with L-NNA treatment and corresponding decrease in nutrient supply to the tumor suggests that autophagy may also contribute. Autophagy under stress is considered a damage limitation program from which cells can escape once “normal” conditions are restored; this may account for why the growth of the tumor is delayed and not halted. Previous studies have also established that autophagy radiosensitizes some tumor cells [31], [32].

The L-NNA-induced decrease in tumor blood flow observed in Figure 4 results in increased tumor hypoxia when measured by pimonidazole staining, the gold standard in assays for tumor hypoxia *in situ*. We also stained tumor sections for endogenous markers of hypoxia, HIF-1α and CA-9. Both these markers did not respond to L-NNA treatment which is not that surprising given their low dynamic response range to changes in tumor oxygen levels. For example, HIF-1α is stabilized at oxygen concentrations as high as 6% [33]. Consequentially large areas of untreated tumor will stain positive for both HIF-1α and CA-9. Endogenous markers are also sensitive to microenvironmental changes independent of oxygen concentration, e.g. NO [34]. That CA-9 is also non-responsive to L-NNA treatment probably reflects the fact that HIF-1α regulates CA-9 expression [35].

Endothelial cell generated NO mediates relaxation of vascular smooth muscle and thus, in normal tissues, inhibition of eNOS can cause hypoxia by decreasing vasodilation and enhancing vasoconstriction [36], [37]. Because of their disorganized and poorly functional vasculature, most tumors have regions of intermittent hypoxia that contain viable cells. However persistent inhibition of endothelial cell eNOS with continuous exposure to a NOS inhibitor can convert a state of intermittent hypoxia to one that is chronic with the consequences very different from acute, intermittent hypoxia. In evaluating mechanisms underlying the effects of chronic hypoxia on cell activities investigations have been confined to *in vitro* analyses to permit defined levels of oxygenation. These studies have demonstrated that *in vitro* proliferation of tumor cells is unhindered by chronic hypoxic conditions as low as 0.2% oxygen [19]. Our own unpublished studies with A431 and FaDu cells have confirmed this. However, expression of homologous recombination DNA repair proteins is partially suppressed in some cells chronically exposed to low oxygen. There is a corresponding decrease in error free DNA repair and corresponding radiosensitization and sensitization to DNA damaging drugs [19]. This possibly provides one explanation for why chronic hypoxia, in contrast to acute hypoxia, also radiosensitizes cells [18], [19]. However, we were unable to demonstrate in *in vitro* clonogenic assays using A431 cells radiosensitization using near identical low oxygen levels and length of hypoxia (Figure 5 A & B). The effects of prolonged hypoxia *in vitro* may be cell type specific.

Results from *in vitro* clonogenic assays testing the effects of hypoxia are only indicative of possible changes in oxygen delivery and do not reflect other consequences of reduced blood flow such as the disruption of nutrient delivery and waste disposal which may also contribute to radiosensitization. As shown in previous studies inhibiting glycolysis can be both cytostatic and cytotoxic [38], [39]. 2-deoxyglucose, for example, inhibits cell cycle progression and is cytotoxic to tumor cells especially under hypoxic conditions. Induction of chronic hypoxia may also suggest why L-NNA is cytotoxic *in vivo* but not *in vitro* beyond invoking an exclusively antiangiogenesis mechanism. *In vitro* studies have demonstrated that inhibition of NOS with L-NNA can block important cytoprotective mechanisms such as such as ras/Akt pathways and NF-κB signaling [2], [6], [10]. Blocking these NO-sensitive cytoprotective mechanisms, however, may only result in cell killing under hypoxic stress conditions. This is currently under study.

The administration of L-NNA elicits only a temporary restraint upon tumor growth indicating some compensatory mechanism is activated resulting in resumed tumor growth (Figure 1). In terms of tumor endothelial cell proliferation one such mechanism is an “angiogenic switch” whereby blocking of one angiogenic pathway is compensated by the activation of an alternative angiogenic pathway [40]. Our finding of enhanced arginase II expression with NOS inhibition by L-NNA treatment suggests one such compensatory pathway. We have not, however, observed any increase in CD31 positive staining or structures to accompany increased angiogenesis. This does not preclude an increase in CD31 negative nascent vasculature [41]. Previous studies have established that hypoxia induces expression of arginase II in diverse cell types including endothelial and vasculature smooth muscle cells and that over expression of arginase II stimulates endothelial cell proliferation [27], [42]. There also appears to be a reciprocal relationship between NOS expression and arginase II expression at least in some cell types [26], [42]. The enhanced arginase II expression we observe with prolonged L-NNA treatment may represent a growth promoting compensatory mechanism [28].

Other investigators have tested NOS inhibitors as anti-tumor agents, often with less efficacious results than seen in the present study. For example, L-NNA delivered i.p. 30 minutes prior to 20 Gy IR in mice bearing murine transplantable tumors induced full radiobiological hypoxia and increased tumor cell clonogenicity [43]. L-NAME delivered i.p. 1 hour prior to IR does not enhance a 16 Gy dose in delaying the growth of FsaII murine fibrosarcomas [13]. Similarly, L-NAME added to drinking water blocks an increase in pO_2_ seen 24 hrs post IR, does not enhance the effect of 6 Gy IR in murine transplantable liver tumors (TLT), and abolishes the effect of a second dose of IR [11]. Overnight treatment with L-NAME in the drinking water of FsaII or TLT bearing animals has no effect upon tumor pO_2_ 4 hrs after 4 Gy IR [12]. Our experimental approach differs significantly from many of these studies in that we use L-NNA rather than L-NAME, we treat with L-NNA overnight prior to IR and continue to provide L-NNA for the duration of the experiment. Furthermore, we were not able to replicate our L-NNA data using L-NAME in our model. Not all tumor types appear as responsive to NOS inhibition as squamous carcinomas [13], [44]. NO can act to both promote and inhibit tumor progression and metastasis depending upon NOS activity, isoforms present, concentration and duration of NO exposure and cellular sensitivity to NO [45].

Previous studies have combined IR with therapies targeting specific mechanisms (e.g. angiogenesis [46]) or specific molecular targets (e.g. VEGFR [47]). For the most part these strategies have proven only marginally effective in radiotherapy of head and neck squamous carcinomas [48]. L-NNA represents an agent that acts with minimal side effects but in a more general manner to affect multiple cellular survival mechanisms in both tumors and the supporting stroma. For example, by mitigating the activity of the PI3K-AKT-eNOS Ras pathway, or the NF-κB pathway, NOS inhibition has the potential to affect many components of tumor maintenance and growth [2], [6], [10]. A recent study has shown NOS inhibition to reduce lymph node metastasis from a peripheral ear model of fibrosarcoma [44]. Consideration thus should be given as to how decreased tumor burden following combined treatment corresponds to increased survival. A similar increase in survival does not follow with the decreased tumor burden observed with either L-NNA or IR alone, as such the role of NO in other pathologic processes such as tumor metastasis and cell migration need consideration [45], [49].
